# Simultaneous Bilateral Spontaneous Pneumothorax: A Rare Complication of Osteosarcoma

**DOI:** 10.7759/cureus.2745

**Published:** 2018-06-05

**Authors:** Usman Tariq, Muhammad Saad Sohail, Zainab Fatima, Amina Khan, Abu Baker Sheikh, Shimron I Bhatti

**Affiliations:** 1 Research Assistant, Yale University School of Medicine, New Haven, USA; 2 Internal Medicine, Shifa International Hospital, Islamabad, PAK; 3 Medicine, Shifa International Hospital, Islamabad, PAK; 4 Shifa Tameer E Millat University, Shifa International Hospital, Islamabad, PAK; 5 Internal Medicine, Shifa International Hospital, Islamabad, Select Country; 6 Orthopaedics, Shifa International Hospital, Islamabad, PAK

**Keywords:** pneumothorax, bilateral pneumothorax, osteosarcoma, pulmonary metastasis, rare complication

## Abstract

The lungs are a common site of metastatic spread of an osteosarcoma. An affiliated simultaneous bilateral spontaneous pneumothorax (SBSP) is a rare clinical sequela of this malignancy. In this case report, we present the clinical circumstances of a young teenager who presented to our clinical setting following a diagnosis of osteosarcoma. We also illustrate the postulated pathophysiology, the tools for diagnosis and a subsequent management for this rare clinical entity.

## Introduction

Osteosarcoma is the most common primary bone malignancy in children and assumes the eighth rank in a list of the most frequently occurring malignancies among the pediatric population [1]. Metastatic spread to the lungs is a common dilemma with this malignancy, as up to 20% of the patients present with a pulmonary dispersion at the time of their diagnosis, while as many as 60% of the patients have subclinical micro-metastasis at the same time [2]. The annexation of the lung parenchyma by a metastatic spread of a malignancy can lead to spontaneous pneumothorax (SP).

Simultaneous bilateral spontaneous pneumothorax (SBSP) accounts for 1.3 to 1.9 percent of all cases of SP and is usually associated with underlying malignancies such as Hodgkin's lymphoma, lymphangioleiomyomatosis, mesotheliomas, and osteosarcomas with pleural and/or parenchymal invasion [3].

In this case report, we present the case of a 16-year-old-male with a diagnosed left fibular osteosarcoma in conjunction with pulmonary metastasis who developed the rare complication of SBSP.

## Case presentation

**Figure 1:**
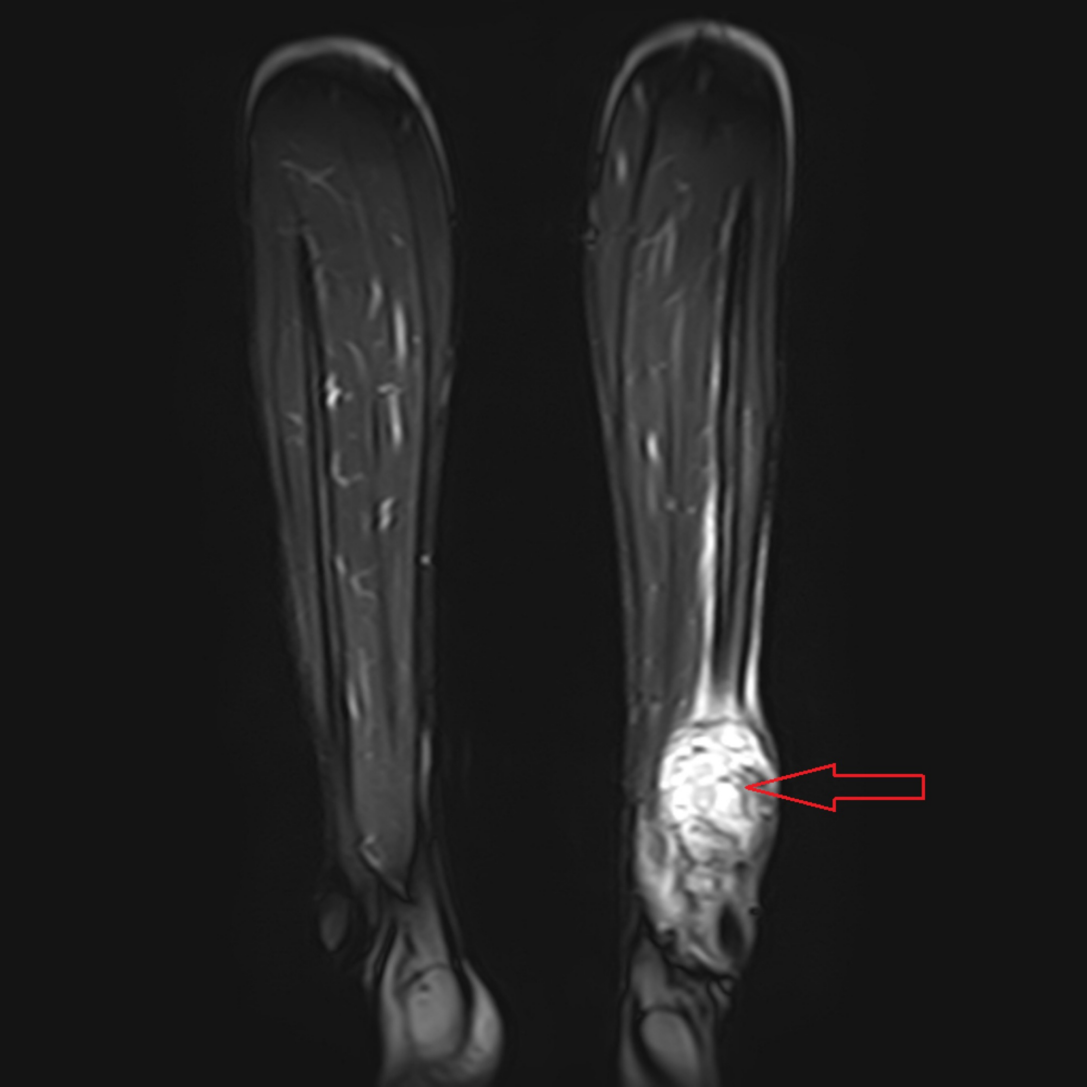
Contrast-enhanced magnetic resonance imaging (MRI) scan of the lower extremities. Red arrow shows a well-defined multicystic mass arising from the distal metaphyseal region of the left fibula.

A 16-year-old male, prior to his presentation at our clinical setting, was diagnosed with an osteosarcoma in his left fibula. At the time of the diagnosis, he presented with a painful and progressively enlarging mass over the left ankle which restricted any weight bearing on the affected leg. A contrast-enhanced magnetic resonance imaging (MRI) scan of the lower extremities revealed a well-defined multicystic mass arising from the distal metaphyseal region of the left fibula (Figure 1).

**Figure 2:**
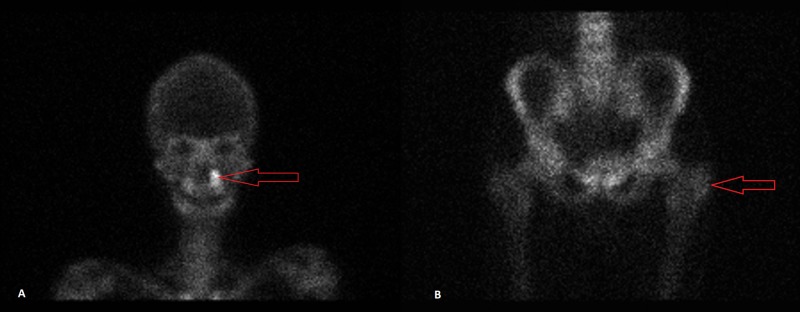
Bone scan. Image A: Red arrow points toward increased tracer uptake in the left maxilla. Image B: Red arrow points toward mildly increased tracer uptake in the greater trochanter of the left femur (compare with right femur).

The mass was subjected to a core needle biopsy and subsequent histological evaluation of the biopsy specimen revealed a collection of hypercellular, spindle polygonal cells with an abundance of osteoclastic giant cells; which provided the tissue diagnosis of an osteosarcoma. Following this diagnosis, the patient presented to our clinical setting for further management. He underwent surgical excision of the tumor followed by the placement of a vascularized fibular bone graft. Following the surgery, the patient was provided with a total of 32 cycles of adjuvant chemotherapy with cisplatin, methotrexate, and leucovorin. He subsequently went into remission. After six months, he presented again with complaints of resurfaced pain in his left leg. A bone scan was performed due to the suspicion of tumor recurrence, which revealed an intense and irregular uptake in the distal segment of the left leg; confirming the reemergence of his primary pathology. The bone scan also showed areas of bony metastasis (evidenced by multiple areas of moderate tracer uptake) in the left maxilla, left parietal bone of the skull and greater trochanter of the left femur (figure 2).

**Figure 3:**
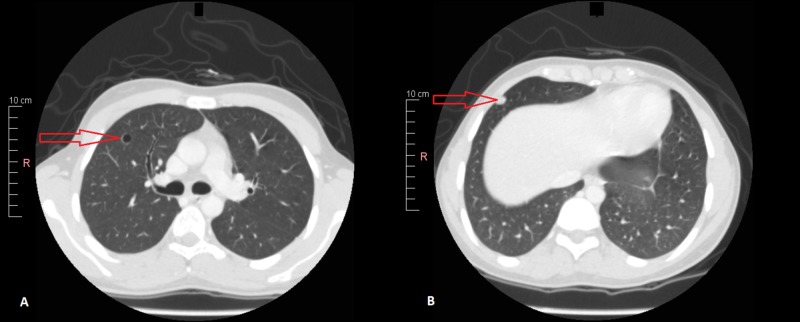
Contrast-enhanced high resolution computed tomography (HRCT) scan of the chest. Image A: Red arrow points toward a cystic cavity in the right upper lobe. Image B: Red arrow points toward a pleural-based nodule (likely metastatic).

**Figure 4:**
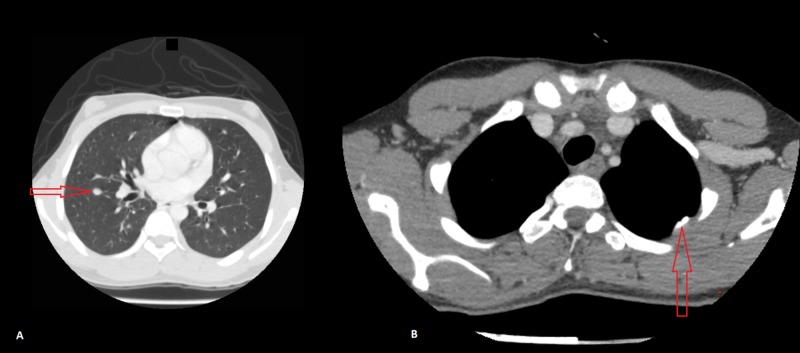
Contrast enhanced high resolution computed tomography (HRCT) scan of the chest. Image A: Red arrow points toward a calcified nodule in the right lung (likely metastatic). Image B: Red arrow points toward a pleural-based nodule (likely metastatic).

Further assessment with a contrast-enhanced high-resolution computed tomography (HRCT) scan of the lungs revealed multiple soft tissue nodules of differing sizes in both lungs. Some of these nodules were pleural-based and some showed internal cavitations (with the largest in the right upper lobe measuring 1.8 cm in diameter), which were suggestive of a metastatic disease process (Figure 3, Figure 4).

**Figure 5:**
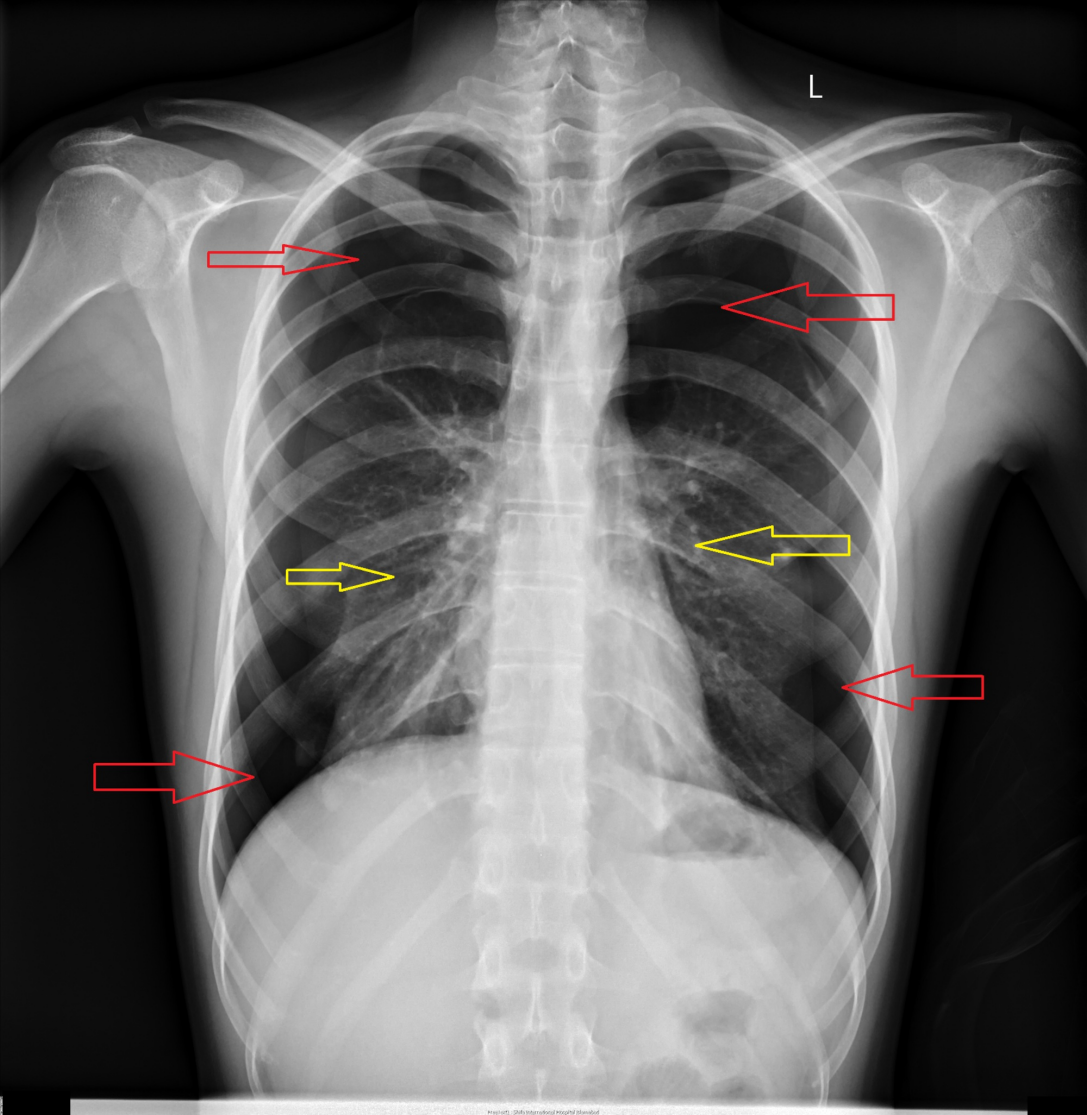
Chest X-ray showing bilateral pneumothorax. Red arrows point toward highly radiolucent areas in the thorax demonstrating bilateral pneumothorax. Yellow arrows point toward medially displaced lung parenchyma due to bilateral pneumothorax.

The patient was subsequently started on a second-line chemotherapeutic regimen, which comprised of etoposide, ifosfamide, and mesna. One week (and two cycles) into his chemotherapy, the patient presented with complaints of shortness of breath (SOB) that particularly worsened on exertion. His predicament was accompanied by pleuritic chest pain and an intermittent, dry cough. The initial assessment revealed a blood pressure (BP) of 140/100 mmHg, heart rate (HR) of 115/minute, respiratory rate (RR) of 25/minute and temperature of 37.2 ˚C (98.96 ˚F). He was alert and well-oriented with a Glasgow Coma Scale (GCS) score of 15/15. Examination of the chest showed a hyper-resonant percussion note, and auscultation revealed bilaterally decreased breath sounds. Further evaluation with a chest X-ray revealed bilateral pneumothoraces with medial displacement of the lung parenchyma on both sides (Figure 5).

**Figure 6:**
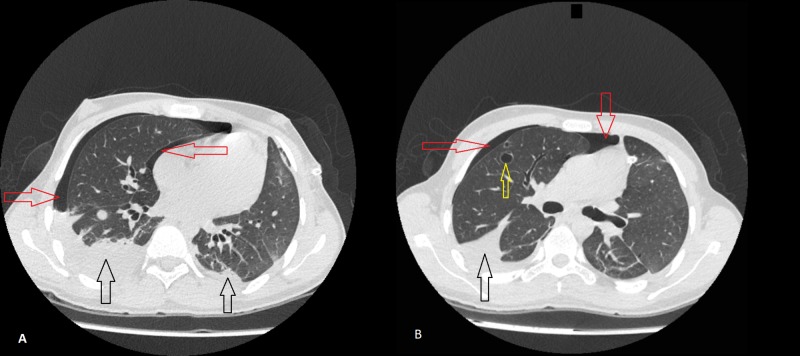
Computed tomography (CT) scan of the chest without contrast. Image A: Red arrows point toward pneumothorax in the right lung; black arrows point toward bilateral pleural effusions. Image B: Red arrows point toward pneumothorax in the right lung; black arrow points toward a pleural effusion; yellow arrow points toward a cystic lesion (1.8 cm) in the upper lobe of the right lung.

An electrocardiogram (ECG) showed sinus tachycardia and an echocardiogram revealed an ejection fraction of 60%. Chest tubes (with an underwater air seal) were inserted bilaterally. During the course of this admission, the patient received multiple chest X-rays to follow the status of his pneumothoraces. These X-rays showed a marked reduction in the volume of the left pneumothorax but only minimal improvement of the right pneumothorax. A computed tomography (CT) scan of the chest without contrast showed bilateral pneumothoraces with a right-sided prominence as well as numerous cystic lesions in both lung fields; with the largest in the right upper lobe (1.8 cm in diameter) (Figure 6).

**Figure 7:**
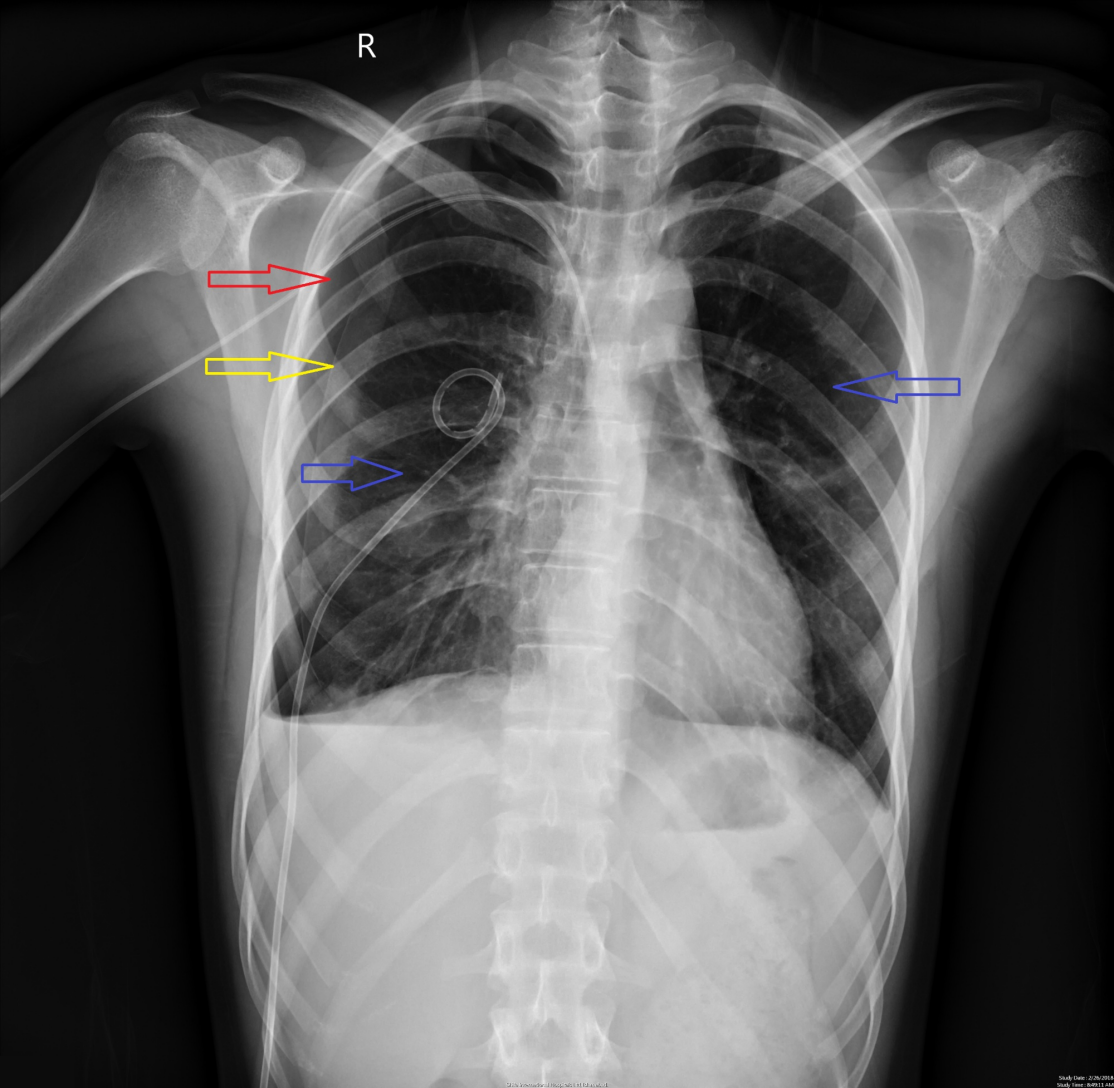
Chest X-ray showing resolving pneumothorax bilaterally. Red arrow points toward an area of radiolucency in the right thorax indicating a partially resolved pneumothorax. Yellow arrow points toward the beginning of the lung parenchyma on the right side indicating a partial pneumothorax on the right side. Blue arrows point toward normal lung parenchyma.

A lack of improvement following a bilateral chest tube insertion incited a subsequent chemical (talc) pleurodesis, which yielded significant clinical and radiological improvement. He was later discharged with a right pigtail insertion. A follow-up chest X-ray revealed the resolution of the bilateral pneumothoraces (Figure 7) and his chemotherapy was resumed.

**Figure 8:**
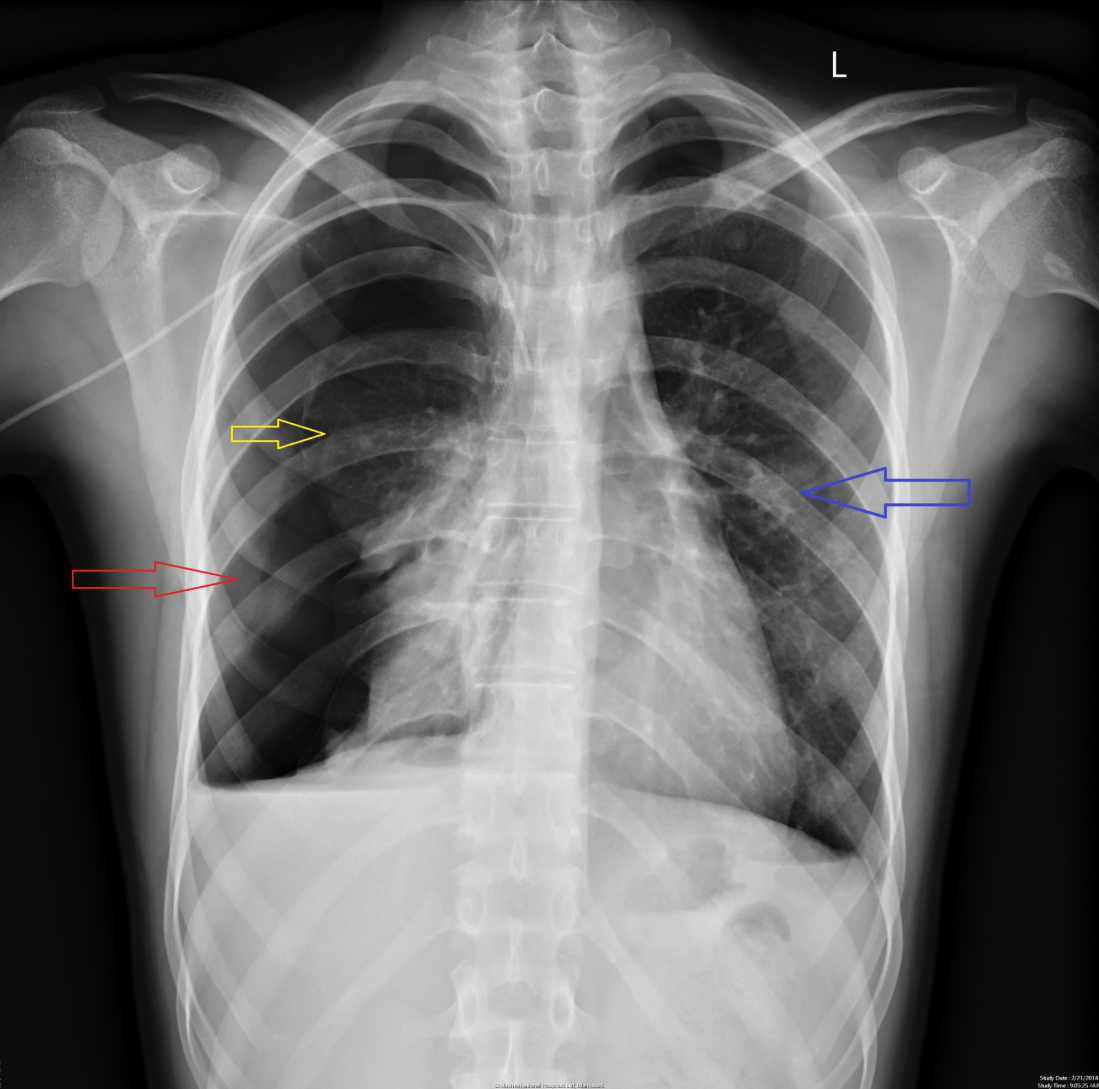
Chest X-ray showing recurrent pneumothorax on the right side. Red arrow points toward an extensive area of radiolucency on the right side signifying right sided pneumothorax. Yellow arrow points toward medially displaced lung parenchyma on the right side. Blue arrow points toward normal lung on the left side.

The patient was readmitted to our clinical setting after one month following the resolution of his bilateral pneumothoraces; this time with a recurrent right-sided pneumothorax (Figure 8).

A second chemical (talc) pleurodesis could not be performed owing to the patient’s refusal for the procedure, while a video-assisted thoracoscopic surgery (VATS) was not offered because of the widespread pulmonary metastatic lesions. In this admission he was managed conservatively via a chest drain, ultimately resulting in the resolution of the pneumothorax.

## Discussion

Epidemiology and survival

Metastatic spread to the lungs is a well-documented complication of an osteosarcoma. Up to 50% of the patients afflicted with this malignancy develop pulmonary metastasis; either as a synchronous or metachronous growth [4]. However, the development of a concomitant spontaneous pneumothorax is not very common, with less than 2% of the patients developing collapse as a consequence of the metastatic disease process [5]. This underscores the importance of having a high degree of suspicion in patients with osteosarcoma who present with complaints of chest pain and shortness of breath.

The survival rate of patients with metastatic osteosarcomas is regrettably low, with a five-year survival rate between 10 to 30 percent [6]. Dismayingly, the development of spontaneous pneumothorax as a subsequent complication also mounts to the impairment of patients’ quality of life and their survival. Less than 10% of osteosarcoma patients survive beyond a two-year limit following the diagnosis of a pneumothorax [7].

Pathogenesis

Various mechanisms have been implicated to explain the pathophysiology of a spontaneous pneumothorax in patients with malignancies. Metastatic nodules close to the bronchial lumen may cause an impediment of airflow, resulting in the entrapment of air in the alveoli in a ball-valve mechanism. The resultant enlargement in the alveolar cavity may lead to a break in the thin alveolar wall, causing the air to escape via the interalveolar planes into the overlying pleura. This may lead to the development of subpleural blebs that can rupture, leading to a spontaneous pneumothorax. The tumor nodule may initially form close to the boundary of a previously formed cystic cavity, such as in patients with previous chronic obstructive pulmonary disease (COPD), pulmonary tuberculosis (TB), asthma or bronchiectasis. The wall of the cavity might break down secondary to tumor invasion, leading to its subsequent disintegration and an open channel into the pleural cavity [8].

Mezghani et al. reported a case of spontaneous pneumothorax in a patient with osteosarcoma without any metastatic involvement of the lungs [9]; which alludes to factors other than the physical presence of a metastatic tumor nodule. Regardless of the presence of widespread metastatic involvement of the lungs, patients who are subjected to chemotherapy for an osteosarcoma are more prone to develop a spontaneous pneumothorax. According to Smevik et al., the introduction of chemotherapy in patients with osteosarcoma increases their risk of developing spontaneous pneumothorax from 7% to 14% [10]. Various mechanisms have been considered to explain how a spontaneous pneumothorax develops after chemotherapy. Rupture of a subpleural and/or emphysematous bleb, and tumor necrosis and lysis secondary to the cytotoxic action of the chemotherapeutic drugs leading to the development of a fistulous tract are some of the working theories used to explain this association [11].

In our patient, the pneumothorax occurred after the formation of cystic lesions in his lungs. We can hypothesize that the pneumothorax developed secondary to these lesions. In lieu of this assumption, we can attribute the severe right sided pneumothorax to the large persistent cystic cavity in his right upper lobe (figure 6). In addition, our patient developed his first episode of pneumothorax after the initiation of the second-line chemotherapeutic regimen (to treat his osteosarcoma recurrence) that included Ifosfamide and Etoposide, which could also be a possible inciting factor for the pneumothorax.

Diagnosis and management

Chest computed tomography (CT) scan is considered to be the gold standard for the diagnosis of a pneumothorax. It has a much higher sensitivity in comparison to a chest X-ray (CXR). In one study, the pooled sensitivity and specificity of CXR was shown to be 31.8% and 100% (respectively) when compared to a chest CT. The CT scan has the ability to visualize the chest in a three-dimensional capacity, which enables it to detect an SP of any size. A CXR has its limitations because it can miss a SP that could be detected on a chest CT. This type of SP is defined as an occult SP in modern literature. Another limitation of a CXR is establishing a diagnosis of a SP in a patient lying supine. In this setting, a relatively large SP (which usually requires emergent treatment) may not be properly visualized, which may result in an unfavourable outcome. However, a CT scan can be more productive in terms of diagnosing such forms of SP [12].

Treatment

Initial management of bilateral spontaneous pneumothorax includes hospitalization and administration of supplemental oxygen. The decision to insert a chest tube is determined by the etiology and size of the pneumothorax. Pneumothoraces larger than 1-2 cm in size, or in the setting of an underlying lung disease such as lung metastasis or COPD require chest drain insertion. Additional interventions are required in the case of a persistent or recurrent pneumothorax. These include surgical strategies or chemical pleurodesis. Surgical interventions include video-assisted thoracoscopic surgery (VATS) or open thoracotomy. Both procedures entail a resection or stapling of the blebs followed by a chemical or surgical pleurodesis. A chemical pleurodesis involves the instillation of a sclerosing agent such as tetracycline in the pleural space. This induces a localised inflammatory reaction; leading to the formation of adhesions which causes the closure of the pleural space. However, chemical pleurodesis alone is less effective than VATS and should only be performed in individuals who are either unable or unwilling to undergo surgery [13].

## Conclusions

Simultaneous bilateral spontaneous pneumothorax is a rare but potentially fatal complication of an osteosarcoma. This is especially true for patients with metastatic lung lesions or those receiving chemotherapy. Consequently, a clinician needs to have a high level of suspicion for pneumothorax if the patient presents with symptoms like dyspnea and pleuritic chest pain. A pulmonary involvement may result in persistent and/or recurrent bouts of pneumothoraces that may require multiple admissions. This can complicate the management of the primary pathology and impair the patient’s quality of life, resulting in increased morbidity and mortality.
